# Supplementary figures and images for: Development of an optimized protocol for generating knockout cancer cell lines using the CRISPR/Cas9 system, with emphasis on transient transfection
Source: PLoS One. 2024 Nov 14;19(11):e0310368. doi: 10.1371/journal.pone.0310368 (PMC11563393; doi:10.1371/journal.pone.0310368)

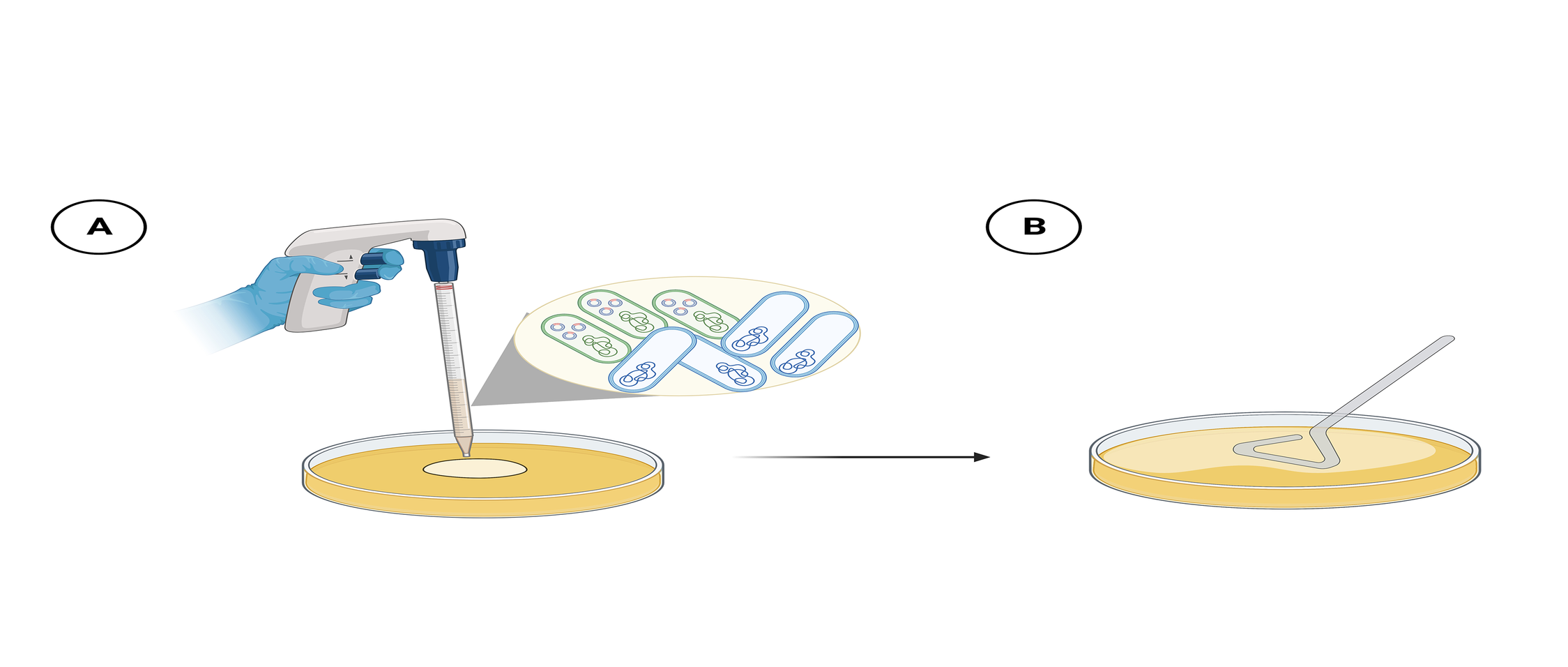

Supplement: S1 Fig — A) Pouring homogenized suspension onto LB-Agar plate: 1) Prepare LB-agar plates containing 100 μg/mL ampicillin. 2) Homogenize bacterial suspensions for both the transformed cells and negative control. 3) Carefully pour each homogenized bacterial suspension into the center of the LB-agar plate, ensuring a gentle pour to prevent splashing or uneven distribution. B) Evenly Distributing the Suspension Using a Sterile Disposable Spreader: 4) Pour onto the agar plate using a sterile disposable spreader following suspension. 5) Start from the center where the suspension was poured; gently move the spreader back and forth to distribute the bacterial suspension evenly at the agar’s surface. Maintain a gentle and smooth spreading motion to prevent damage to the agar surface or establish uneven bacterial colonies. Created with BioRender.com. (TIF) [file pone.0310368.s001.tif]

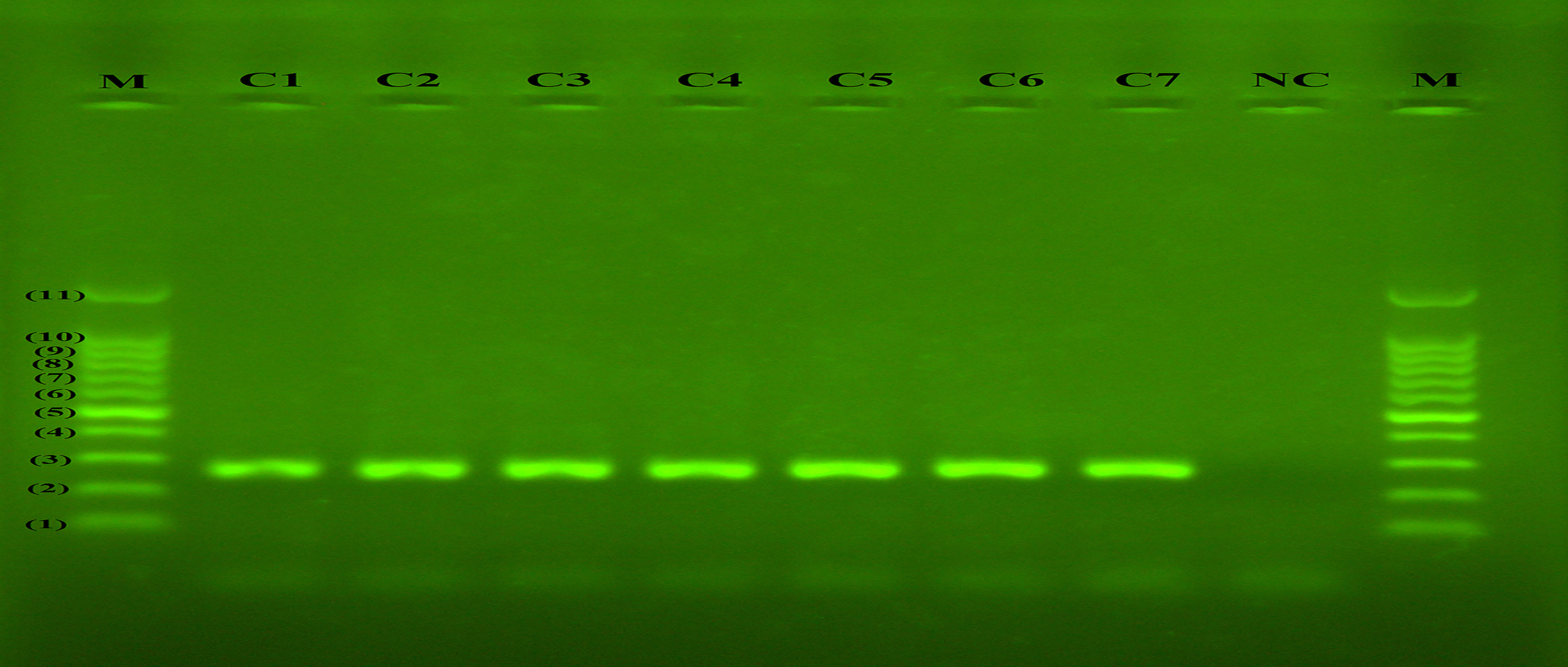

Supplement: S2 Fig — Each PCR product exhibits a distinct 273-bp band, confirming the accurate cloning of the sgRNA. The term ’NC’ denotes the negative control, showing no bands. A ladder reference, ranging from 100 to 1500 bps, was employed, with bands separated by 100-bp intervals. Notably, the highest band appears at 500 bps beyond the 1000-bp band. This outcome strongly supports the successful sgRNA cloning process within the recombinant pX459 plasmids. (TIF) [file pone.0310368.s002.tif]

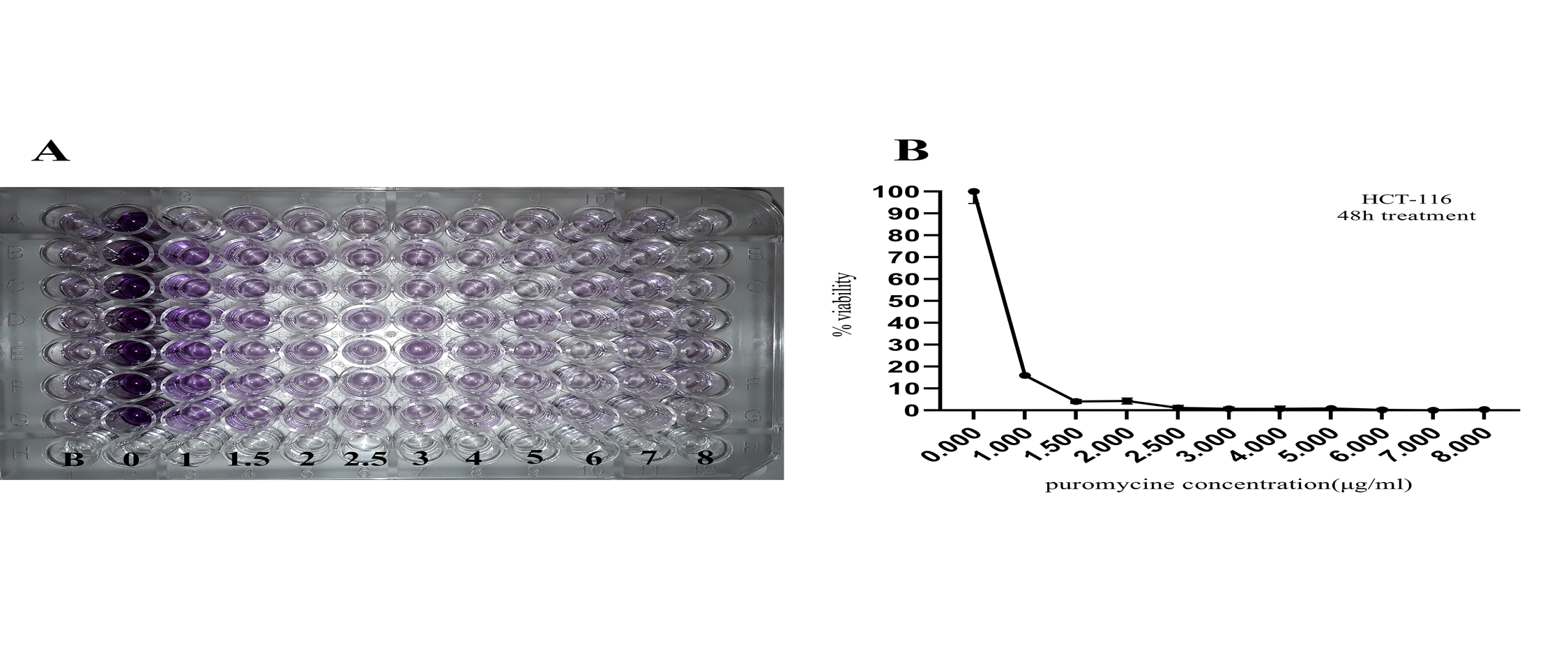

Supplement: S3 Fig — A) 96-Well Plate for the ELISA absorbance measurements. Each column represents distinct concentrations of puromycin antibiotic treatment ranging from 0 to 8 μg/ml. Specific concentrations are labeled at the column base, while ’B’ denotes the blank value. A stronger purple coloration indicates a high level of living cells. B) Puromycin kill curve. We measured absorbances at 570 nm and 630 nm for seven replicates of each concentration of puromycin treatment. The results can be found in the S6 and S7 Tables. We imported the final raw data (provided in S8 Table) into GraphPad Prism for analysis and to draw the puromycin kill curve for the HCT-116 cell line, which received a 48-hour puromycin treatment. The graph shows a negative correlation between puromycin concentration and cell viability. The desired concentration is defined as the lowest amount of puromycin at which cell viability reaches zero. Our results showed that 3 μg/ml puromycin is the minimum concentration required to achieve complete death of untransfected cells. This chart also includes standard deviation values; for some concentrations, the standard deviation is less than 1 percent, making it difficult to detect on this scale. For concentrations such as 0 μg/ml (negative control) and 2 μg/ml, which exhibit higher standard deviations, detection is easier. All survival percentages and corresponding standard deviations for each concentration are listed in the S9 Table. (TIF) [file pone.0310368.s003.tif]

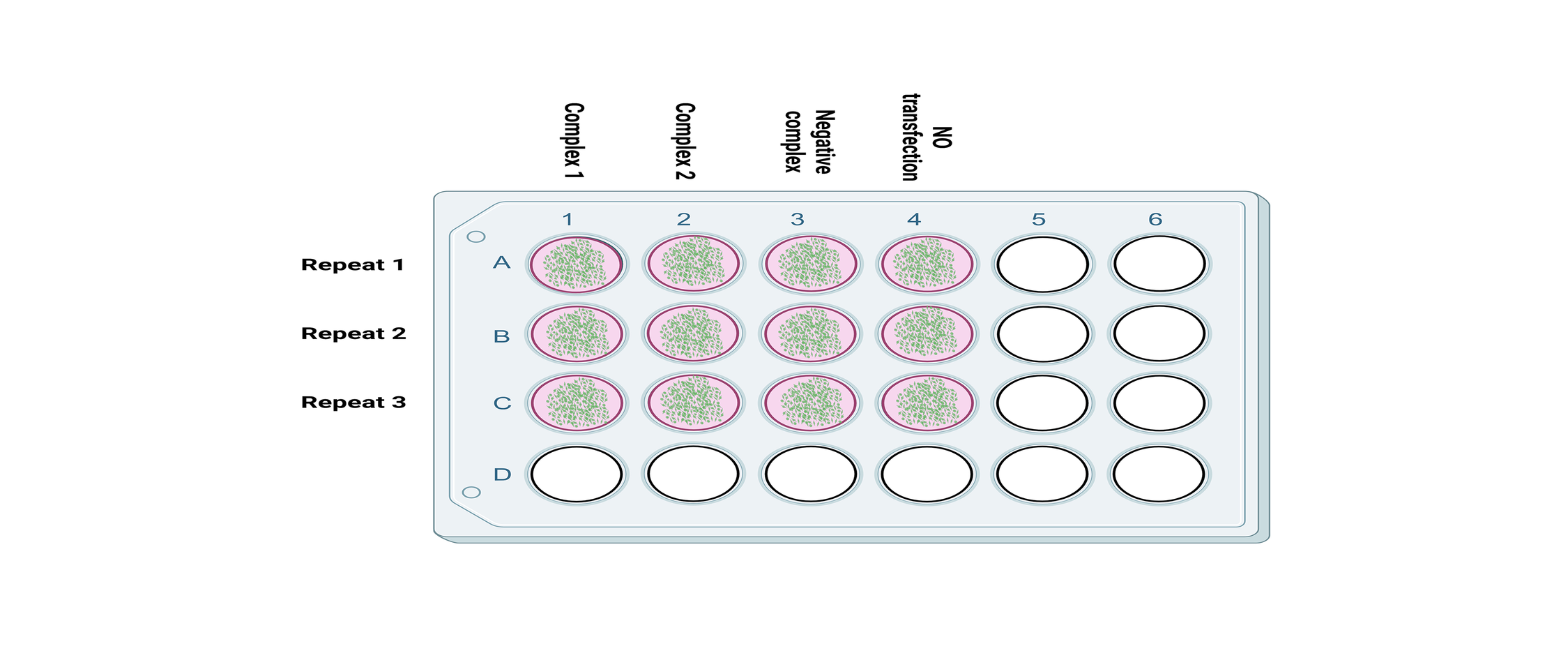

Supplement: S4 Fig — Columns 1 and 2 contain three replicates of Complex-1 and Complex-2, respectively. Column 3 represents the negative control, devoid of a recombinant plasmid and containing only lipofectamine. This control group is used to evaluate the probable side effects of lipofectamine during transfection. Column 4 represents the null transfection control, untreated with any complex. This control is used to verify the accuracy of puromycin selection after transfection. The control groups (negative control and null transfection) are placed in separate columns to facilitate easy comparison and analysis of the experimental results. Created with BioRender.com. (TIF) [file pone.0310368.s004.tif]

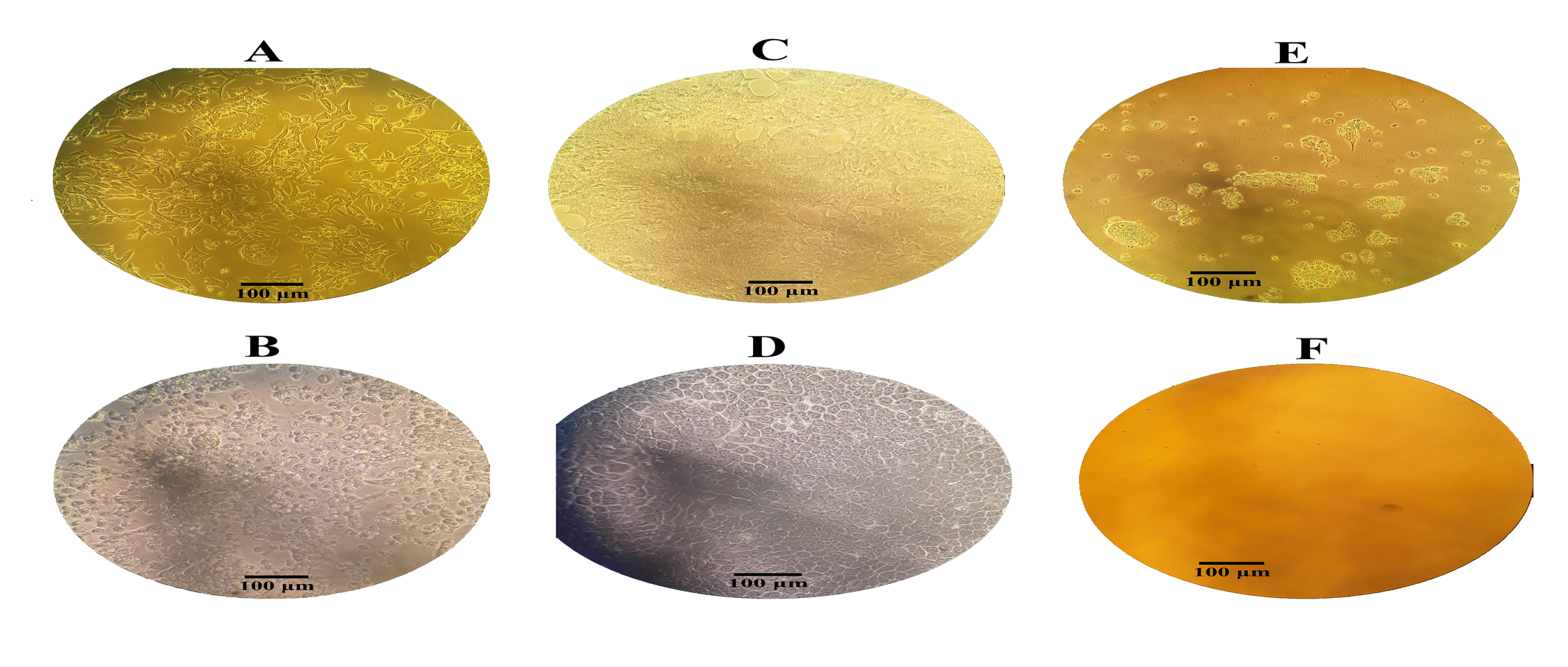

Supplement: S5 Fig — A) At the initiation of transfection, HCT-116 cells demonstrate a confluence of 50–60%. B) At 48 hours post-transfection, cells treated with Complex-1 exhibit reduced elongated shapes compared to their initial state. C) At 48 hours post-transfection, cells treated with negative-complex display a remarkable increase in confluence, reaching 90–100%. Dense cell junctions are prominently observed. D) Cells in the no-transfection well reach complete confluence 48 hours post-transfection. All observations were obtained using a 20× objective lens. E) After a 48-hour puromycin selection, the complex-1 well displays numerous transfected cells. F) Conversely, cells in the no-transfection well after 48-hour puromycin selection exhibit complete cell death. Visualizations for (E) and (F) were obtained using a 10× objective lens. (TIF) [file pone.0310368.s005.tif]

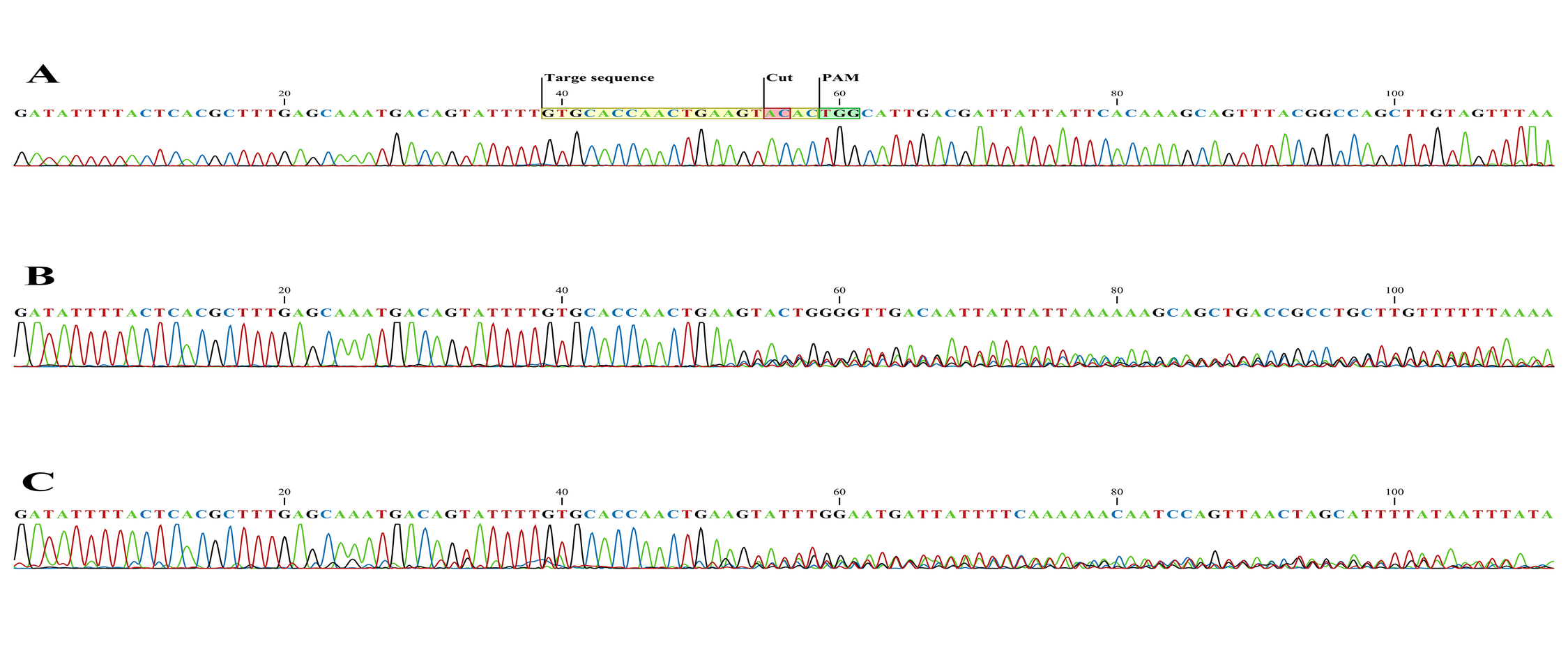

Supplement: S6 Fig — A) The sequencing result of the HCT-116 wild-type population illustrates the regular and unique peaks of sequence arrangement. The PAM and the 20-nt target sequences are highlighted in green and yellow, respectively. The cleavage site is located between nucleotides A and C, which is marked in red. B, C) Images B and C indicate the sequencing results of cell populations treated with complex-1 and complex-2, respectively. The NHEJ mechanism induces different repairs at the cleavage sites, resulting in varied sequences between cells. Consequently, multiple nucleotide variations emerge at unique locations, manifesting as irregular short peaks in the sequencing data. In contrast to the wild-type population containing both the PAM and the 20-nt target sequence, which are concurrently present, these sequences are not simultaneously identified within populations treated with complex-1 and complex-2. Overall, this result provides a comprehensive visual of the correct performance of the CRISPR system, elucidating the genetic modifications in complex-1 and complex-2 compared to the wild-type population. (TIF) [file pone.0310368.s006.tif]

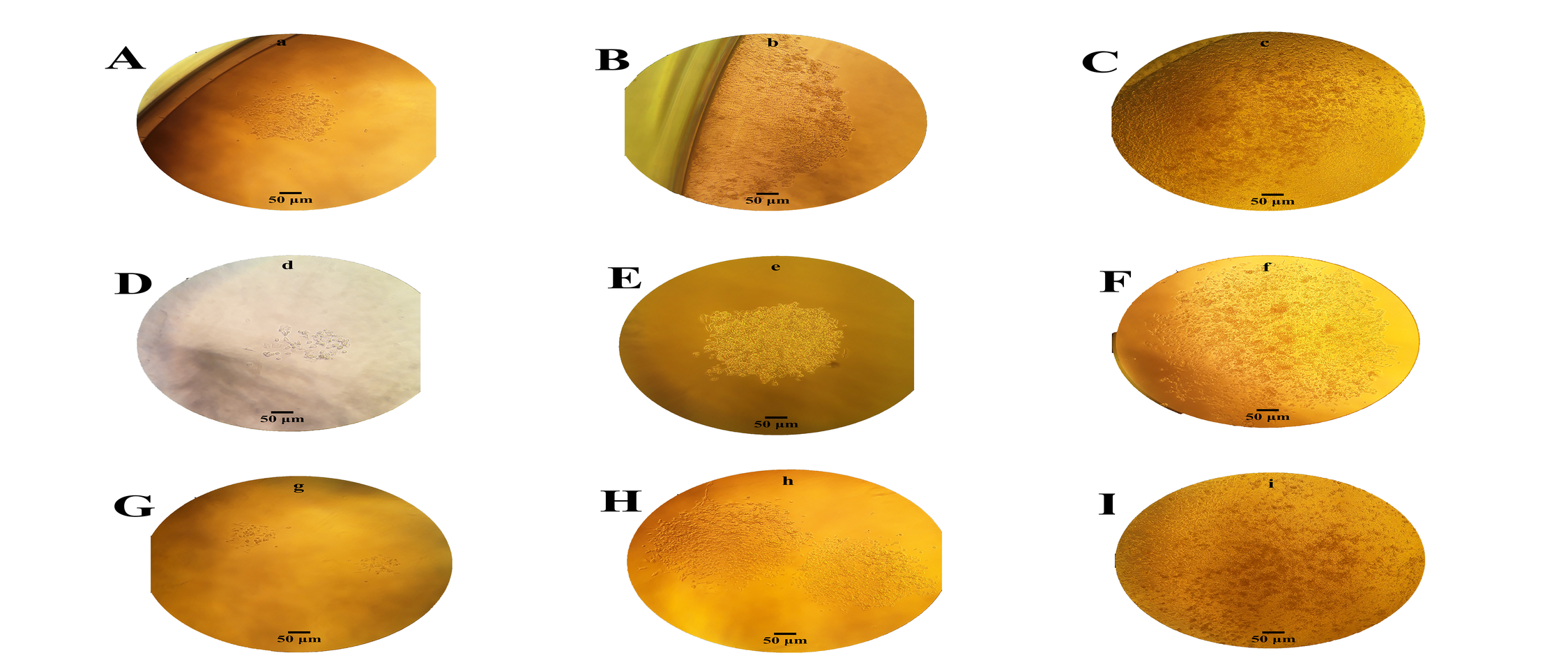

Supplement: S7 Fig — A, B, C) Z-14 single cell population. Images A, B, and C exhibit the Z-14 single-cell population after 7, 14, and 21 days of isolation, respectively. This single cell was attached to the corner of the well after isolation. D, E, F) X-2 single cell population. Images D, E, and F showcase the X-2 single-cell population after 7, 14, and 21 days of isolation, respectively. Similar to the Z-14 population, these images illustrate the progression of single-cell proliferation over the three weeks. G, H, I) X-7 Cell Population. Images G, H, and I present the X-7 cell population after 7, 14, and 21 days, respectively. Notably, the X-7 well initially received two cells during the isolation process. These images vividly depict the high proliferation of population due to originating from a multi-cellular state. All images were captured using a 10x objective lens. (TIF) [file pone.0310368.s007.tif]

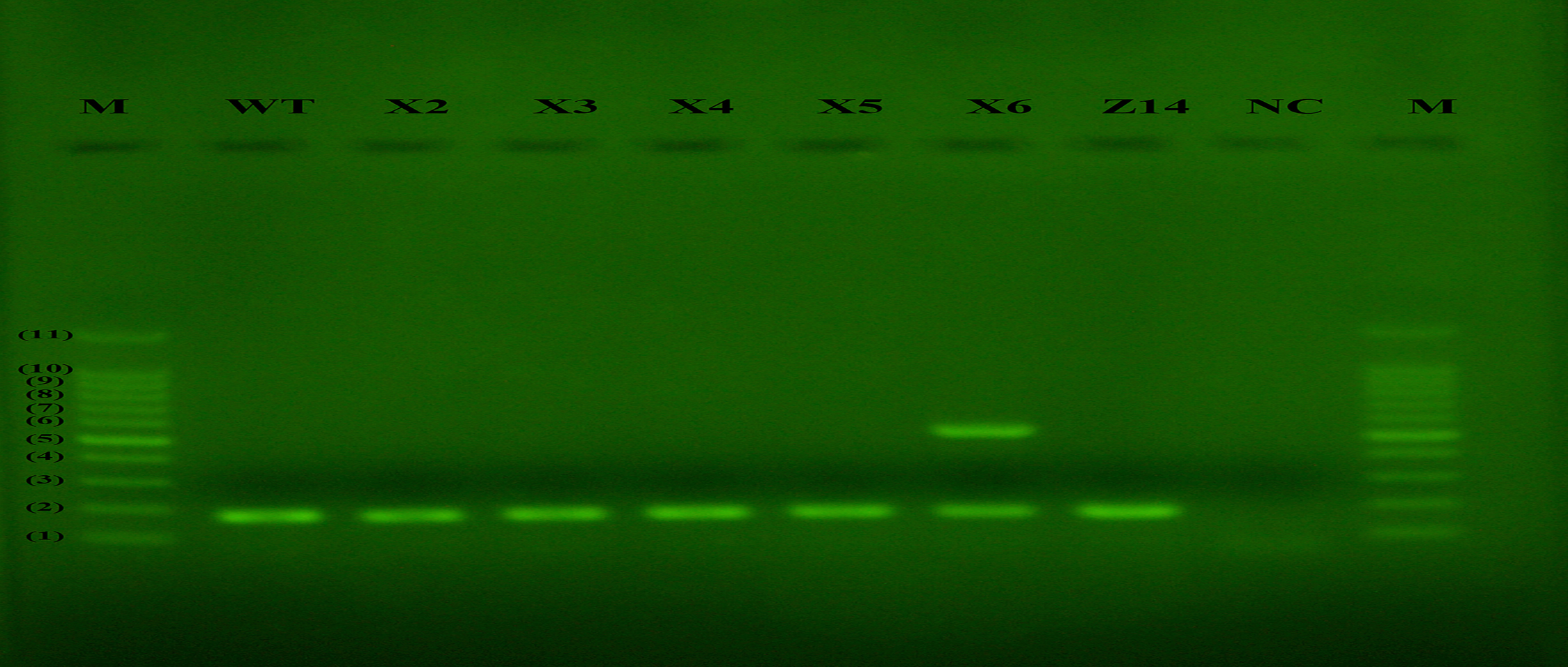

Supplement: S8 Fig — Performing gel electrophoresis is necessary to evaluate PCR product quality for Sanger sequencing. All samples, except for X6, exhibited a singular band at around 180 bps. The X6 sample displayed an additional band ranging between 500 and 600 bps. We purified both bands from the gel and executed Sanger sequencing. The insertion of a fragment from the plasmid containing the Cas9 coding region into the cleavage site of one allele was validated by further analysis. Notably, ’NC’ denotes the negative control, showing no bands. A 100–1500 bps ladder was utilized, with bands spaced 100 bps apart, while the highest band was 500 bps distant from the 1000 bps band. (TIF) [file pone.0310368.s008.tif]
