# Supplementary material for: Development of an optimized protocol for generating knockout cancer cell lines using the CRISPR/Cas9 system, with emphasis on transient transfection
Source: PLoS One. 2024 Nov 14;19(11):e0310368. doi: 10.1371/journal.pone.0310368 (PMC11563393; doi:10.1371/journal.pone.0310368)
Supplement: S1 File — (PDF) [file pone.0310368.s009.pdf]

Step-by-Step Protocol for Generating CRISPR-Mediated Knockout Cancer Cell Lines Using Transient Transfection

Seyed Alireza Mousavi Kahaki, Nayereh Ebrahimzadeh, Hossein Fahimi, Arfa Moshiri

PART 1: Designing an effective gRNA

1d

1

Note

Utilize the following five online databases to design the 20-nt target sequence for the gene of interest (GOI) based on the criteria described in the '**Experimental Design**' section of the manuscript. Arrange the identified gRNAs according to the S2 Table.

CHOPCHOP

The ranking of designed gRNAs will prioritize those with the fewest off-target effects based on default settings. Selected gRNAs must not induce any harmful off-target effects. Additionally, the tool suggests multiple primers for each gRNA to amplify the target fragment. Mismatches in the predicted off-target are indicated in red and lowercase letters.

Software

CHOPCHOP

NAME

CITATION

Labun K, Montague TG, Krause M, Torres Cleuren YN, Tjeldnes H, Valen E (2019). CHOPCHOP v3: expanding the CRISPR web toolbox beyond genome editing..

LINK

https://doi.org/10.1093/nar/gkz365

2

CRISPOR

The tool's output categorizes results into three colors, with green indicating the highest specificity and efficiency. The most likely off-targets are those shown in grey, which have no mismatches in the seed region.

Software

CRISPOR NAME

CITATION

Concordet JP, Haeussler M (2018). CRISPOR: intuitive guide selection for CRISPR/Cas9 genome editing experiments and screens..

LINK

https://doi.org/10.1093/nar/gky354

3 **E-CRISP**

The ranking of designed gRNAs is based on the specificity, annotation, and efficiency (SAE) score. "Green" denotes suitable gRNAs, while "Red" flags potentially harmful ones.

Software

E-CRISP NAME

German Cancer Research Center DEVELOPER

CITATION

Heigwer F, Kerr G, Boutros M (2014). E-CRISP: fast CRISPR target site identification..

LINK

https://doi.org/10.1038/nmeth.2812

4 **BENCHLING**

The designed gRNAs are ranked based on two parameters: firstly, the on-target activity score, adjusted according to Doench, Fusi et al., and secondly, the off-target specificity score, adjusted according to Hsu et al.. gRNAs with values closer to 100 for both metrics are considered superior. Each off-target score number selection presents the probability of off-target activity, detailing the percentage and position. One of the notable advantages of this software is its accessibility to multiple transcript types of the GOI.

Software

Benchling [Biology Software] NAME

Benchling DEVELOPER

CITATION

Doench JG, Fusi N, Sullender M, Hegde M, Vaimberg EW, Donovan KF, Smith I, Tothova Z, Wilen C, Orchard R, Virgin HW, Listgarten J, Root DE (2016). Optimized sgRNA design to maximize activity and minimize off-target effects of CRISPR-Cas9..

LINK

https://doi.org/10.1038/nbt.3437

CITATION

Hsu PD, Scott DA, Weinstein JA, Ran FA, Konermann S, Agarwala V, Li Y, Fine EJ, Wu X, Shalem O, Cradick TJ, Marraffini LA, Bao G, Zhang F (2013). DNA targeting specificity of RNA-guided Cas9 nucleases..

LINK

https://doi.org/10.1038/nbt.2647

5 **SYNTHEGO**

This advanced tool empowers scientists to select highly specific and efficient gRNAs. Unlike other software, this tool suggests a limited number of gRNAs by simultaneously considering multiple crucial parameters for sgRNA design. These include the presence in exons present in all transcripts of the gene, positioning within the earliest coding exons, and a high predicted efficiency in inducing InDels. However, notable limitations of this tool include a lack of support for different endonucleases, an unalterable PAM sequence, and its inapplicability to other CRISPR applications such as knock-in, activation, and repression.

Software

CRISPR Design Tool
NAME

Synthego
DEVELOPER

6 Select a gRNA present in all gRNA design tools and it has achieved the highest ranking. The gRNA selected in our study is highlighted in grey in the S2 Table.

Note

TROUBLESHOOTING (See S1 Table)

7 Design and order the sgRNA-top and sgRNA-bottom oligonucleotides with 5'-end phosphorylation (Approach 1) or without 5'-end chemical modification (Approach 2).

Note

Be cautious about the PAM sequence! The *Streptococcus pyogenes* Cas9 (SpCas9) PAM sequence (NGG) is related to the Cas9 endonuclease enzyme and should not be added to the 20-nt target sequence.

PART 2: Preparation of insert fragment for cloning

1d

8

Note

There are two approaches for preparing the insert fragment. If Approach 1 (Using 5'-end phosphorylated oligonucleotides) is chosen, skip Approach 2 (Treatment of oligonucleotides without chemical modification by the T4PNK enzyme) (STEP 9 to STEP 10), and proceed with the procedure from STEP 11.

Approach 1 (STEP 8)
Resuspend sgRNA-top and sgRNA-bottom oligonucleotides, 5'-end phosphorylated (from STEP 7), in ddH_2O to create a 100 µM stock solution (Store at -20 °C). Then, as outlined in the following table, prepare a 10 µM concentration of each oligonucleotide in a 0.2 ml PCR microtube. Mix gently, then spin briefly.

Component	Amount
sgRNA-top (sgRNA-top-EpCAM) (100 µM)	2 µl
sgRNA-bottom (sgRNA-bottom-EpCAM) (100 µM)	2 µl
ddH2O, DNase free	16 µl
Total	20 µl

9 **Approach 2** (STEP 9 to STEP 10)

Resuspend sgRNA-top and sgRNA-bottom oligonucleotides, without chemical modification of the 5'-ends (from STEP 7), in ddH_2O to create a 100 µM stock solution (Store at -20 °C). Perform the phosphorylation of the 5'-ends of the oligonucleotides using T4 PNK by preparing a 10 µM concentration of each oligonucleotide in a 0.2 ml PCR microtube, as outlined in the following table. Mix gently, then spin briefly.

Component	Amount
sgRNA-top (sgRNA-top-EpCAM) (100 µM)	2 µl
sgRNA-bottom (sgRNA-bottom-EpCAM) (100 µM)	2 µl
T4 PNK	10 U
T4 Ligation Buffer 10X	2 µl
ddH2O, DNase free	Up to 20 µl
Total	20 µl

10 Incubate the reaction mixture from STEP 9 in a thermocycler at 37 °C for 30 minutes to complete phosphorylation. Then, increase the temperature to 75 °C and incubate the microtube for 10 minutes to inactivate the T4PNK enzyme.

11 Set the thermal cycler according to the continuous gradual cooling program outlined in the following table to execute the 'Insert self-annealing' step for the products obtained in either STEP 8 or STEP 10. If your thermal cycler doesn't support such gradual cooling, skip this step and use the alternative program provided in STEP 12.

Step	Temperature	Time	Cycle number
Denaturation	95 °C	5 minutes	1
Cool down	−0.1 °C/s until 25 °C	-	1
Hold	25 °C	15 minutes	1
Finish	4 °C	-	-

12 Execute the 'Insert self-annealing' process for the products obtained in STEP 8 or STEP 10 by configuring the thermal cycler with the specified program, gradually reducing the temperature from 95 to 25 °C in short intervals.

Temp	Time	Temp	Time	Temp	Time	Temp	Time
95 °C	5 Min	77 °C	20 Sec	59 °C	20 Sec	41 °C	20 Sec
93 °C	20 Sec	75 °C	20 Sec	57 °C	20 Sec	39 °C	20 Sec
91 °C	20 Sec	73 °C	20 Sec	55 °C	20 Sec	37 °C	20 Sec
89 °C	20 Sec	71 °C	20 Sec	53 °C	20 Sec	35 °C	20 Sec
87 °C	20 Sec	69 °C	20 Sec	51 °C	20 Sec	33 °C	20 Sec
85 °C	20 Sec	67 °C	20 Sec	49 °C	20 Sec	31 °C	20 Sec

Temp	Time	Temp	Time	Temp	Time	Temp	Time
83 °C	20 Sec	65 °C	20 Sec	47 °C	20 Sec	29 °C	20 Sec
81 °C	20 Sec	63 °C	20 Sec	45 °C	20 Sec	27 °C	20 Sec
79 °C	20 Sec	61 °C	20 Sec	43 °C	20 Sec	25 °C	15 Min

Temp: Temperature
Min: Minutes
Sec: Seconds

Note

Take care! STEP 11 and STEP 12 produce identical results. Please select one of the two steps for "Insert self-annealing" based on the thermal cycler's capabilities.

13 Dilute the Insert fragment solution (STEP 11 or STEP 12) to a 1:100 ratio in a 0.5 ml microtube to obtain the ready-to-use insert solution. Ensure thorough pipetting to homogenize the solution and then briefly spin.

Component	Amount
Insert segment solution (STEP 11 or STEP 12)	2 µl
ddH2O, DNase free	198 µl
Total	200 µl

PART 3: Digestion and ligation reactions and creation of the recombinant plasmid

2d

14

Note

Approach 1 involves separate digestion, ligation, and gel purification steps, while Approach 2 combines digestion and ligation without gel purification. If you select Approach 1, skip Approach 2 (STEP 20 to STEP 23), continuing directly from STEP 24.

Approach 1 (STEP 14 to STEP 19)

Digest 1µg of the pX459 vector with the FD-BbsI enzyme in a 0.5 ml PCR microtube according to the following table. Mix gently, then spin briefly.

Component	Amount
pX459 vector (1 µg)	X µl
10X Fast digest buffer	2 µl
FastDigest BbsI enzyme	1 µl
ddH2O DNase free	Up to 20 µl
Total	20 µl

Note

Ensure that the volume of the FD-BbsI enzyme does not exceed 10% (vol/vol) of the final reaction volume. This precaution is necessary as reagents in the FD-BbsI enzyme storage buffer, such as glycerol, may interfere with the digestion reaction.

15 Incubate the digestion reaction (from STEP 14) in a thermocycler at 37 °C for 30 minutes to complete digestion. Subsequently, raise the temperature to 65 °C and incubate for 10 minutes to deactivate the FD-BbsI enzyme.

Note

Based on the enzyme data sheet, the FD-BbsI enzyme demonstrated no star activity throughout the 16-hour digestion period. However, this protocol extends the digestion reaction's incubation time to 30 minutes to ensure complete linearization of all plasmids.

16 Use fresh TAE buffer to create a 0.6-0.8% (wt/vol) agarose gel of adequate thickness. Load 20 µl of the digested product (from STEP 15) into one well and 3 µl of a 1kbs ladder into another. Perform gel electrophoresis at 75 volts for 40 minutes.

Note

TROUBLESHOOTING (See S1 Table)

Note

Due to the inhibitory effect of borate in Tris/Borate/EDTA (TBE) buffer on various enzymes, including ligase, and its potential interference with the ligation reaction, we strongly recommend conducting electrophoresis of the digestion product using fresh TAE buffer.

Note

Low-percentage agarose gels are extremely fragile, and a portion of them melt at temperatures above 30 °C, causing interferences during the cutting process. Consequently, gel transfer and cutting should be executed below 20 °C.

17 Cut the single band located between 9-10 kbs as quickly as possible under UV irradiation in such a way as to harvest the lowest possible gel mass. Purify the linearized pX459 band according to the gel purification kit manufacturer's instructions and elute the column with 25 µl of the kit's elution buffer to recover the maximum concentration of linearized pX459. Then, measure the concentration of it using a Nanodrop spectrophotometer.

Note

TROUBLESHOOTING (See S1 Table)

18 Perform the ligation reaction between the purified linearized pX459 from the gel (STEP 17) and the insert fragment (STEP 13) in a 0.5 ml PCR microtube at an approximate ratio of 1:10 (vector: insert) according to the following table. Mix gently, then spin briefly.

Component	Ligation reaction	NC
Purified linearized pX459 from the gel (200 ng) (STEP 17)	X µl	X µl
Insert segment (from STEP 13)	3.5 µl	-
10X Ligase buffer	2 µl	2 µl
T4 ligase enzyme	1 µl	1 µl
ddH2O, DNase free	Up to 20 µl	Up to 20 µl
Total	20 µl	20 µl

NC: Negative Control

Note

Use the T4 ligation buffer when the white precipitate (containing ATP) in the 10X ligase buffer has completely dissolved.

19 Incubate the ligation reactions (from STEP 18) in a thermocycler at 22 °C for 3 hours. Continue incubation at 16 °C for a minimum of 18 hours. Lastly, raise the temperature to 65°C and incubate for 10 minutes to deactivate the ligase enzyme.

20 **Approach 2** (STEP 20 to STEP 23)
Simultaneously digest and dephosphorylate 1 µg of the pX459 vector using the FD-BbsI enzyme and the FastAPTM Thermosensitive Alkaline Phosphatase in a 0.5ml PCR microtube, according to the following table. Mix gently, then spin briefly.

Component	Amount
pX459 vector (1 µg)	X µl
10X Ligase buffer	2 µl
FastDigest BbsI enzyme	1 µl
FastAP™ Thermosensitive Alkaline Phosphatase	1 µl
ddH2O DNase free	Up to 20 µl
Total	20 µl

21 Incubate the reaction (from STEP 20) in a thermocycler at 37 °C for 30 minutes to complete the digestion and dephosphorylation reactions. Inactivate the digestion and FastAPTM enzymes by raising the temperature to 80 °C and incubating for 20 minutes.

Note

If the enzymes are not inactivated correctly in this step, the yield of the desired ligation reaction will be drastically reduced.

22 Perform the ligation reaction between the linearized pX459 (STEP 21) and the insert fragment (STEP 13) in a 0.5 ml PCR microtube at an approximate ratio of 1:10 (vector: insert), as indicated in the following table. Mix gently, then spin briefly.

Component	Ligation reaction	NC
Linearized pX459 (final product from STEP 21)	4 µl	4 µl
Insert segment (from STEP 13)	3.5 µl	-
10X Ligase buffer	2 µl	2 µl
T4 ligase enzyme	1 µl	1 µl
ddH2O, DNase free	9.5 µl	13 µl
Total	20 µl	20 µl

NC: Negative Control

Note

Use the T4 ligation buffer when the white precipitate (containing ATP) in the 10X ligase buffer has completely dissolved.

23 Incubate the ligation reactions (STEP 22) in a thermocycler at 22 °C for 3 hours. Continue incubation at 16 °C for a minimum of 18 hours. Lastly, raise the temperature to 65°C and incubate for 10 minutes to deactivate the ligase enzyme.

PART 4: Treatment with plasmid-safe endonuclease (optional)

1d

24 Treat the ligation reaction products (from either STEP 19 or STEP 23) with a plasmid-safe endonuclease, according to the following table, to remove any residual linearized DNA.

Component	Ligation reaction	NC
Ligation reaction product (STEP 19 or STEP 23)	20 µl	20 µl
10X Reaction Buffer	3 µl	3 µl
25 mM ATP	2 µl	2 µl
Plasmid-Safe DNase (10 U)	1 µl	1 µl
ddH2O, DNase free	4 µl	4 µl
Total	30 µl	30 µl

NC: Negative Control

Note

Ensuring the complete dissolution of the white precipitate before utilizing the ATP solution is crucial.

25 Incubate the reactions at 37 °C for 1 hour using a thermal cycler, then raise the temperature to 70 °C and continue incubation for 30 minutes to deactivate the plasmid-safe endonuclease enzyme.

PART 5: Bacterial transformation and recombinant plasmid extraction

2d

26 Remove the required amount of chemically competent DH5 alpha cells from -70 °C and allow them to thaw on ice for 10 minutes. Use 200 µl of competent cells for 20 µl of ligation product or 30 µl of the plasmid-safe treatment product.

27 Add the ligation products (or the plasmid-safe treatment products) to a microtube containing chemically competent DH5-alpha cells. Perform separately for the ligation reaction and negative control, and then incubate on ice for 40 minutes. Remove the microtubes from the ice every 10 minutes and gently tap the bottom of the microtubes several times to homogenize the competent cells and recombinant plasmid, and then place the microtubes back on ice.

28 Incubate two microtubes in a thermoblock set to 42°C for 1 minute for heat shock, followed by immediate placement back on ice for a 10-minute incubation.

29 Transfer the contents of the two microtubes into separate sterile 50 ml conical tubes containing 750 µl of pre-warmed LB Broth medium at 37°C without ampicillin. Incubate the conical tubes at 37°C and 125 RPM on a shaker for 1 hour.

30 Transfer the contents from 50 ml canonical tubes into new sterile 1.5 ml microtubes and centrifuge them at 13000 RPM for 10 minutes. Remove and discard 700 µl of the supernatant, then utilize the remaining supernatant to prepare a homogenized suspension of the white pellet.

31 Remove the homogenized suspension separately from the microtubes with the pipette and pour it over the center of two LB-agar plates containing 100 µg/ml ampicillin. Employ a sterile disposable spreader to distribute the suspension evenly across the plates. Incubate the plates at 37°C for 18 hours.

Note

TROUBLESHOOTING (See S1 Table)

32 Using the sterile, disposable pipette tips, select multiple colonies and introduce them into sterile 50 ml conical tubes containing 10 ml of LB medium with 100 µg/ml ampicillin. For the negative control, insert an empty (colony-free) sterile, disposable pipette tip into a 50 ml sterile conical tube containing 10 ml of LB medium with 100 µg/ml ampicillin. For the

positive control, transfer one colony using a sterile, disposable pipette tip to a 50 ml sterile conical tube containing 10 ml of LB medium without ampicillin.

33 Incubate the bacterial cultures in a shaking incubator at 37 °C and 125 RPM for 18 hours. Your results must align with the following outcomes.

Expected result

Sample	Growth	Transparency	Result
Negative control	No	Transparent LB-Broth medium	There is no contamination.
Positive control	Yes	Turbid LB-Broth medium	The cultivation process is performed correctly.
Transformed bacteria	Yes	Turbid LB-Broth medium	Only transformed bacteria have grown.

34 Take an appropriate volume of the culture medium according to the plasmid purification kit manufacturer's instructions to extract the recombinant pX459 plasmid. Subsequently, measure the concentration of the extracted plasmid using a Nanodrop spectrophotometer.

35 Prepare the following requisite reagents for the PCR to validate the accuracy of recombinant pX459 plasmid generation before initiating the transfection process. Mix gently, then spin briefly.

Component	Colony Z	NC
Extracted recombinant Plasmid (10 ng) (from STEP 34)	X µl	-
hU6 forward primer (10 µM)	1 µl	1 µl
sgRNA-bottom-EpCAM (Reverse primer) (10 µM)	1 µl	1 µl
Pfu DNA Polymerase MasterMix [2X]	12.5 µl	12.5 µl
ddH2O	Up to 25 µl	10.5 µl
Total	25 µl	25 µl

NC: Negative Control

36 Use the following program to perform the PCR reactions in STEP 35:

Step	Number of cycles	Temperature	Time
Primary denaturation	1 X	95 °C	00:05:00
Denaturation	25 X	95 °C	00:00:35
Annealing	25 X	55-60 °C	00:00:35
Extension	25 X	72 °C	00:00:30
Final extension	1 X	72 °C	00:03:00
Hold	1 X	4 °C	-

37 Load a suitable volume of the PCR product reactions from Step 36 into each well of a 1.5% agarose gel, conducting electrophoresis at 80 volts for 45 minutes. Each reaction must display a 273-bp band, except for the negative control, which will exhibit no band.

Note

TROUBLESHOOTING (See S1 Table)

PART 6: Performing the MTT assay to determine optimal puromycin concentration

4d

38 Prepare a 10 ml cell suspension using DMEM containing 10% FBS, including 3×10^5 cells per ml. Employ a multichannel pipette to dispense 100 µl of the cell suspension into each well of a 96-well plate, resulting in a density of approximately 3×10^4 cells per well. Seed 77 wells according to the S4 Table, ensuring the first column and last row remain vacant. Post-incubation for 24 hours, expect complete adherence of cells to the well surfaces.

39 According to the S3 Table, combine the determined volumes of puromycin solution (W_A or W_B) with fresh DMEM containing 10% FBS to create 11 distinct concentrations, each with a final volume of 1 ml in sterile 1.5 ml microtubes. Vortex the microtubes for 2 seconds, then briefly spin. Considering the photosensitivity of puromycin, execute all procedures away from direct light exposure within the laminar hood.

Note

Instead of 6, 7, and 8 µg/ml, utilize concentrations of 0.25, 0.5, and 0.75 µg/ml when preparing the various concentrations for suspension cell lines.

40 Remove and discard the existing culture medium from each well. Then, according to the S4 Table, add 100 µl of the culture medium containing the respective puromycin concentrations prepared in Step 39 to each well. Incubate the plate for 48 hours.

41 Dispense 100 µl of the 5 mg/ml MTT solution into 11 sterile 1.5 ml microtubes following the guidelines in S5 Table. Add the specified volume of puromycin solution from W_A or W_B, and then add the fresh DMEM containing 10% FBS to achieve a final volume of 1 ml in each microtube. Vortex the microtubes for 2 seconds, then briefly spin. Due to the light sensitivity of both puromycin and MTT solutions, perform all procedures away from direct light exposure within the laminar hood.

42 Remove and discard the existing culture medium from each well. Then, according to the S4 Table, add 100 µl of the culture medium containing the MTT and puromycin solutions prepared at STEP 41 to each well. Incubate the plate for 4 hours.

43 Remove and discard the existing culture medium from each well. Add 100 µl of DMSO to each well, pipetting gently to dissolve the formazan precipitate. Cover the entire plate with aluminum foil and shake it at room temperature (RT) for 15 minutes using a microplate shaker. Immediately following, measure the absorbance at 570 and 630 nm.

Note

Fill all wells in column 1 with 100 µl of DMSO to establish blank values for measuring absorbance at two wavelengths. This step allows for subtracting background noise from the main absorbance measurements.

44 Utilize GraphPad Prism to calculate the final results and draw the puromycin kill curve. Select the lowest concentration at which the average survival rate reaches zero. For further assistance, refer to Tables S6, S7, S8, and S9.

Software

Prism NAME

GraphPad DEVELOPER

Note

TROUBLESHOOTING (See S1 Table)

PART 7: Transfection of the recombinant plasmid into the cell line

6d

45 Seed 12 wells of a 24-well plate with approximately 1.2×10^5 cells per well, using a total volume of 700 µl of DMEM containing 20% FBS, according to the pattern outlined in S4 Fig. It is necessary for cells to completely attach to the wells following an overnight incubation.

46 Dilute 0.75 µl and 1.25 µl of Lipofectamine^{TM} 3000 Reagent by adding 24.25 µl and 23.75 µl of Opti-MEM^{TM} Medium for each well, respectively. According to the following table, prepare three replicates for each dilution and negative control. Vortex microtubes for 3 seconds and then spin briefly. Incubate at RT for 15 minutes.

Component	RXN1 (A)	RXN2 (A)	NC (A)
Lipofectamine 3000 Reagent	2.25 µl	4.5 µl	2.25 µl
Opti-MEM Medium	72.75 µl	70.5 µl	72.75 µl
Total	75 µl	75 µl	75 µl

NC: Negative Control

47 Dilute 500 ng of plasmid by adding 1 µl of P3000^{TM} Reagent and an appropriate volume of Opti-MEM^{TM} medium up to 25 µl final volume for each well. According to the following table, prepare dilutions for six wells in 2 separate microtubes and three replicates for negative control. Label the final dilutions RXN1(B), RXN2(B), and Negative control(B). Mix gently, then spin briefly. Do not vortex. Incubate at RT for 15 minutes.

Component	RXN1 (B)	RXN2 (B)	NC (B)
Plasmid (1500ng)	X µl	X µl	-
P3000™ Reagent	3 µl	3 µl	3 µl
Opti-MEM™ Medium	Up to 75 µl	Up to 75 µl	72 µl
Total	75 µl	75 µl	75 µl

NC: Negative Control

48 Prepare the lipofectamine-DNA complexes and the negative control complex by combining the RXN dilutions from Step 46(A) with those from Step 47(B). Homogenize the mixtures thoroughly by pipetting and incubate the complexes at RT for 30 minutes. Add fresh DMEM containing 20% FBS without penicillin-streptomycin to each complex and mix the complexes by pipetting to homogenize well. Remove and discard the existing culture medium from each well and gently wash the wells with DPBS(1X). According to S4 Fig, slowly pour 500 µl of each complex into the wells. Incubate the plate for 48 hours.

Component	Complex 1	Complex 2	Negative complex
Dilution A (STEP 46)	RXN1(A)	RXN2(A)	Negative control (A)
Dilution B (STEP 47)	RXN1(B)	RXN2(B)	Negative control (B)
Total	150 µl	150 µl	150 µl
Incubate at RT for 30 minutes			
DMEM containing 20% FBS	1350 µl	1350 µl	1350 µl
Total	1500 µl	1500 µl	1500 µl

Note

Preceding the transfection process, thorough decontamination of the incubator and laminar hood is imperative, given the absence of penicillin-streptomycin antibiotics during transfection. Equipment contamination poses a significant risk to cell cultures under these conditions.

49 Prepare the complete DMEM medium containing 1% penicillin-streptomycin and 10% FBS, including the calculated puromycin concentration from Step 44. Remove and discard the existing culture medium from each well. Do not rinse with DPBS(1X). Add 700 µl of prepared medium to each well. Incubate the plate for 24 hours.

50 Refresh the culture medium after 24 hours. Remove and discard the existing culture medium from each well and rinse with DPBS(1X). Add 700 µl of freshly prepared DMEM medium, following the procedure outlined in Step 49, to each well. Continue incubating the plate for 24 hours.

Note

Refreshing the antibiotic-containing culture medium is important. The large number of cells can hinder the efficacy of the remaining antibiotic in media against untransfected cells. Omitting this step may extend the puromycin selection period beyond 48 hours.

51 Utilize an inverted microscope to assess the screening progress. The criterion for screening completion is the absence of viable cells in the negative control and no-transfection wells, indicating complete cell death.

Note

TROUBLESHOOTING (See S1 Table)

Note

If viable cells persist in the negative control and no-transfection wells after 48 hours, remove and discard the existing culture medium from each well and add 700 µl of freshly prepared DMEM medium to each well, similar to STEP 49. Continue incubating the plate for additional hours. Refreshing the medium and extending the incubation time will not significantly affect the transfected cells for several hours but will eliminate untransfected cells.

52 Harvest all replicates (Repeat 1, 2, and 3) of each complex (Complex1 and Complex2) by adding 200 µl of trypsin to each well. Neutralize the trypsin by adding 500 µl of complete DMEM and centrifuge all replicates of each complex in unique sterile 15 ml conical tubes at 1100 RPM for 5 minutes. Remove and discard supernatant and transfer the cells to different T-75 flasks containing a complete DMEM medium with 1% penicillin-streptomycin, 20% FBS without the antibiotic puromycin, and incubate the flasks to increase cell confluency. Refresh the culture medium every 72 hours.

53 When cell confluency reaches 70-80%, employ trypsinization to harvest the cells. Subsequently, divide the cells from each flask into three equal portions. Utilize one portion for genomic DNA extraction following the manufacturer's instructions for the DNA extraction kit. Freeze one portion of the harvested cells as a backup. Finally, expand the remaining portion of cells for subsequent steps, which is isolating monoclonal cells via limiting dilution.

54 Prepare the following requisite reagents for the PCR procedure aimed at amplifying the targeted region for Sanger sequencing analysis of sequence changes within the cleavage site region. Mix gently, then spin briefly.

Component	Wild type	Complex 1	Complex 2	NC
Total genomic DNA (1µg) (from STEP53)	X µl	X µl	X µl	-
Forward primer (EpCAM-F. P) (10 µM)	1.5 µl	1.5 µl	1.5 µl	1.5 µl
Reverse primer (EpCAM R. P) (10 µM)	1.5 µl	1.5 µl	1.5 µl	1.5 µl
Pfu DNA Polymerase MasterMix [2X]	12.5 µl	12.5 µl	12.5 µl	12.5 µl
ddH2O	Up to 25 µl	Up to 25 µl	Up to 25 µl	9.5 µl
Total	25 µl	25 µl	25 µl	25 µl

NC: Negative Control

55 Use the following program to perform the PCR reactions in STEP 54:

Step	Number of cycles	Temperature	Time
Primary denaturation	1 X	95 °C	00:10:00
Denaturation	30 X	95 °C	00:00:45
Annealing	30 X	55-60 °C	00:00:45
Extension	30 X	72 °C	00:00:45
Final extension	1 X	72 °C	00:05:00
Hold	1 X	4 °C	-

56 Load an optimal volume of the PCR product reactions from Step 55 into each well of a 2% agarose gel and run electrophoresis at 80 volts for 1 hour. If long InDels have not occurred during DNA repair, a single band should be seen in all complexes at the predicted site, but no band in the negative control.

Note

TROUBLESHOOTING (See S1 Table)

57 Analyze the Sanger sequencing outcomes using software such as CLC Genomics Workbench, MEGA, or equivalent tools. Wild-type (unedited) samples exhibit high peaks with no background noise. In contrast, edited samples show diverse short peaks at specific locations within the on-target region due to DNA cleavage and varied DNA repair across cells.

Software	
**CLC Genomics Workbench**	NAME
windows 11	OS
Qiagen	DEVELOPER

Software	
**MEGA**	NAME

PART 8: Isolation of monoclonal cell populations by limiting dilution

6w 3d

58 When the expanded cells from Step 53 reach 70–80% confluency, discard the cell culture medium and rinse the cells twice using DPBS(1X). Add 3 ml of trypsin to the flask and allow complete cell detachment. Neutralize the trypsin by adding 3 ml of complete DMEM with 10% FBS, then centrifuge at 1200 RPM for 8 minutes. Discard the supernatant and rinse the cell pellet with DPBS(1X). Centrifuge at 1200 RPM for 8 minutes and discard the supernatant.

59 Add 2 ml of complete DMEM containing 20% FBS and create a homogeneous cell suspension. Employ continuous and gentle pipetting of the cell suspension for 2 minutes, aiming to disrupt as many cell-cell junctions and clumps as feasible. Label this tube as M_1.

60 Create a 1/30 dilution of the cell suspension by combining 100 µl of M_1 with 2.9 ml of complete DMEM containing 20% FBS in a new sterile 15 ml centrifuge tube. Ensure even distribution of cells in the suspension by pipetting. Label this tube as M_2.

61 Prepare a 1/2 dilution by combining 20 µl of M_2 with 20 µl of trypan-blue solution. Load 12-15 µl of this dilution onto a hemocytometer for cell counting. Utilize the provided formula to calculate the total cell count in M_2 (derived from 100 µl of M_1).

$$\textit{Number of cells} = (\textit{Total number of cells in 5 squares})/5 \times 3 \times 2 \times 10^4$$

62 Take a specific volume of M_1 containing 6×10^5 cells and adjust the total volume to 3 ml by adding complete DMEM containing 20% FBS. Employ continuous and gentle pipetting of the cell suspension aiming to disrupt as many cell-cell junctions and clumps as feasible, preventing bubble formation. Label this tube as S_1.

63 Position a sterile 40-µm cell strainer (adjusted according to cell size) atop a new sterile 50 ml centrifuge tube to filter the entire volume of S_1. Take 3 ml of S_1 and evenly pour it vertically onto the filter, applying high pressure for liquid ejection. Label this tube as S_2.

64 Calculate the total cell count in S_2 using the formula outlined in Step 61. Generally, there is a reduction in the overall cell count following filtration. Ensure to factor in the dilution ratio and the volume of cells loaded onto the hemocytometer grid when estimating the total cell count in S_2.

65 Create a 1/100 dilution of S_2 by combining 10 µl of S_2 with 990 µl of complete DMEM containing 20% FBS in a new sterile 1.5 ml tube. Gently pipette to ensure a uniform mixture. Label this tube as S_3.

66 Take a specific volume of S_3 containing 600 cells and add to a complete DMEM containing 20% FBS, adjusting the total volume to 120 ml (achieving a concentration of 5 cells per ml). Label this tube as S_f. Pipette the cell suspension thoroughly for even distribution, then promptly transfer it to a sterile cell reservoir. Use a multichannel pipette to seed ten 96-well plates by dispensing 100 µl of S_f into each well.

Note

Due to the absence of growth factors secreted by neighboring cells, certain cells may exhibit limited viability or proliferation when cultured individually. To overcome this problem, two days before isolating single cells, cultivate the wild-type cells at 20-30% confluency in six T-75 flasks, each containing 12 ml of culture medium. Allow the cells to proliferate in this medium for 48 hours. Subsequently, carefully decant the supernatant to prevent the detachment of viable cells. Centrifuge at 3000 RPM for 15 minutes, remove the culture medium without disturbing the small pellet, and filter using a 0.22-micron filter. Combine 60 ml of the filtered medium with 60 ml of fresh medium to achieve the required culture medium volume for this step.

67 After 10-12 hours, meticulously examine each well of the 96-well plate separately using an inverted microscope. This duration allows most normal adherent cells to adhere to the well bottom but is insufficient for the first cellular division. Wells receive more than a single cell must be marked.

Note

Record the estimated site of cell attachment in each well housing a single cell on a plate map for every 96-well plate. This practice streamlines subsequent evaluations, considering certain cells might adhere to the well corners. The initial examination of a 96-well plate takes approximately 30 to 45 minutes.

68 Incubate the plates for three to four weeks or until reaching 60-70% confluency. Assess the growth rate every three days and evaluate the cell culture medium quality after the first week. If there's a decline in the medium quality, promptly replace it with a fresh similar medium, as the cells are sensitive to their environment at this phase.

Note

TROUBLESHOOTING (See S1 Table)

69 When the isolated cells reach 60-70% confluency, harvest the monoclonal cells by adding 50 µl of trypsin, neutralizing it with 50 µl of complete DMEM, and centrifuging at 1100 RPM for 5 minutes in a sterile 1.5 ml microtube. Remove the supernatant, rinse the cell pellet with DPBS(1X), and centrifuge again at 1100 RPM for 5 minutes. Resuspend the cell pellet in 500 µl of complete DMEM and transfer the suspension to separate T-25 flasks containing a complete DMEM medium containing 1% penicillin-streptomycin and 20% FBS without the antibiotic puromycin. Incubate the flasks to increase cell confluency. Refresh the culture medium every 48 hours.

Note

Be careful! Avoid cross-contamination between the wells containing the single-cell colonies during harvesting.

PART 9: Single cell colony sequencing and knock-out validation

2d

70

Note

In this part, Sanger sequencing should be used to verify the accuracy of the gene knockout in the monoclonal cell populations expanded in STEP 69.

When the monoclonal cell populations reach 80-90% confluency, harvest the cells using trypsinization. Subsequently, divide the cells of each culture flask into two equal fractions. Utilize one fraction for genomic DNA extraction and store the other fraction to maintain the backups of the monoclonal cell population.

71 Prepare the following requisite reagents for the PCR procedure aimed at amplifying the target region for Sanger sequencing and sequence change analysis in the cleavage site region of the monoclonal cell populations. Mix gently, then spin briefly.

Component	Monoclonal cell populations	NC
Total genomic DNA (1µg) (from STEP 70)	X µl	-
Forward primer (EpCAM-F. P) (10 µM)	1.5 µl	1.5 µl
Reverse primer (EpCAM R. P) (10 µM)	1.5 µl	1.5 µl
Pfu DNA Polymerase MasterMix [2X]	12.5 µl	12.5 µl
ddH2O	Up to 25 µl	9.5 µl
Total	25 µl	25 µl

NC: Negative Control

72 Use the following program to perform the PCR reactions in STEP 71:

Step	Number of cycles	Temperature	Time
Primary denaturation	1 X	95 °C	00:10:00
Denaturation	30 X	95 °C	00:00:45
Annealing	30 X	55-60 °C	00:00:45
Extension	30 X	72 °C	00:00:45
Final extension	1 X	72 °C	00:05:00
Hold	1 X	4 °C	-

73 Combine 3 µl of the PCR product reactions from Step 72 with 1 µl of loading buffer. Load this mixture into each well of a 2% agarose gel and conduct electrophoresis at 80 volts for 1 hour. A single band appears for the monoclonal cell populations at the anticipated site if long InDels have not occurred during DNA repair. No band should be visible in the negative control. Directly submit the PCR products for Sanger sequencing.

Note

TROUBLESHOOTING (See S1 Table)

74 Analyze the Sanger sequencing outcomes akin to Step 57. Favorable results entail the control sample showcasing both the 20-nt target sequence and the PAM sequence, characterized by regular high peaks devoid of noise. In contrast, the edited sample should reveal InDels or modified nucleotides at the cleavage site, distinctly differentiating it from the control.

Citations

Step 1

Labun K, Montague TG, Krause M, Torres Cleuren YN, Tjeldnes H, Valen E. CHOPCHOP v3: expanding the CRISPR web toolbox beyond genome editing.
https://doi.org/10.1093/nar/gkz365

Step 2

Concordet JP, Haeussler M. CRISPOR: intuitive guide selection for CRISPR/Cas9 genome editing experiments and screens.
https://doi.org/10.1093/nar/gky354

Step 3

Heigwer F, Kerr G, Boutros M. E-CRISP: fast CRISPR target site identification.
https://doi.org/10.1038/nmeth.2812

Step 4

Doench JG, Fusi N, Sullender M, Hegde M, Vaimberg EW, Donovan KF, Smith I, Tothova Z, Wilen C, Orchard R, Virgin HW, Listgarten J, Root DE. Optimized sgRNA design to maximize activity and minimize off-target effects of CRISPR-Cas9.
https://doi.org/10.1038/nbt.3437

Step 4

Hsu PD, Scott DA, Weinstein JA, Ran FA, Konermann S, Agarwala V, Li Y, Fine EJ, Wu X, Shalem O, Cradick TJ, Marraffini LA, Bao G, Zhang F. DNA targeting specificity of RNA-guided Cas9 nucleases.
https://doi.org/10.1038/nbt.2647
