# Supplementary material for: Development of an optimized protocol for generating knockout cancer cell lines using the CRISPR/Cas9 system, with emphasis on transient transfection
Source: PLoS One. 2024 Nov 14;19(11):e0310368. doi: 10.1371/journal.pone.0310368 (PMC11563393; doi:10.1371/journal.pone.0310368)
Supplement: S1 Table — (DOCX) [file pone.0310368.s017.docx]

**S1 Table.** Comprehensive Troubleshooting

| Step(s) | Problem | Possible reason | Recommended solution |
| --- | --- | --- | --- |
| 6 | **1)** gRNA with predicted harmful off-target effects | **1.1)** The high degree of similarity between the target sequence and the regulatory or coding regions of essential genes | **1.1.1)** Select nCas9 strategy with two gRNAs |
| 16 | **2)** Multiple bands (Plasmid bands) on the agarose gel | **2.1)** Due to a decline in the quality of the BbsI enzyme, the digestion reaction was not performed correctly. | **2.1.1)** Increase the reaction time to 60 minutes, or employ twice as much enzyme. If this does not resolve the problem, try a new enzyme. |
|  | **3)** Smearing in the digested product (no band) | **3.1)** Using a high concentration of restriction enzyme in the digestion reaction | **3.1.1)** Reduce the concentration of the enzyme |
|  |  | **3.2)** Digestion reaction time Extended beyond 30 minutes | **3.2.1)** Reduce the reaction time to 15 minutes |
|  | **4)** The linearized vector's recovery yield is low. | **4.1)** The pores of the column were saturated with agarose gel that had been melted. | **4.1.1)** Cut a smaller piece of agarose gel.  **4.1.2)** Utilize a gel with a reduced thickness or concentration. |
| **17** |  | **4.2)** The primary yellow color (the pH of the binding buffer) altered. | **4.2.1)** As soon as the agarose gel has completely dissolved, 10 µL of 3M sodium acetate at pH 5 must be added to the binding buffer. |
|  |  | **4.3)** The linearized vectors were not efficiently eluted | **4.3.1)** The elution process must be repeated with the pre-warmed previously eluted buffer. Repeat the elution process for the third time. |
|  |  | **4.4)** Purification is done with a poor-quality kit. | **4.4.1)** Purify linearized vector with a high-quality kit from the gel |
| **31** | **5)** No colonies appeared on LB-agar plate after bacterial transformation  **5)** No colonies appeared on LB-agar plate after bacterial transformation | **5.1)** Ligation efficiency was reduced by destroying the sticky end of the linearized vector by prolonged UV irradiation. | **5.1.1)** Reduce the UV intensity during the cutting process and perform the cut as quickly as possible. |
|  |  | **5.2)** The ligation reaction was inhibited by the borate in the TBE buffer, which inhibits enzymatic activity. | **5.2.1)** Use TAE buffer instead of TBE buffer to prevent the inhibitory effect of borate. |
|  |  | **5.3)** Due to an insufficient ratio of vector to insert, ligation yield decreased. | **5.3.1)** The optimal vector-to-insert ratio for this ligation is 1:10; if the desired result is not achieved, increase this ratio to 1:20. |
|  |  | **5.4)** Due to insufficient dissolution of the white ATP precipitate after thawing the ligation buffer or degradation of ATP in the ligation buffer by repeated freeze-thaw cycles, the ligation reaction could be stopped. | **5.4.1)** Mix thoroughly while heating the ligation buffer to 37˚c until the white precipitate disappears. Replace the previous ligation buffer with a new one if ATP degradation is suspected. |
|  |  | **5.5)** Due to a decreased quality of the T4 ligase enzyme, the ligation reaction was not performed properly. | **5.5.1)** employ twice as much enzyme. If this does not solve the problem, try a new enzyme. |
|  |  | **5.6)** Insufficient time and improper temperature for effectively carrying out the ligation reaction. | **5.6.1)** This is a difficult and time-consuming ligation reaction. Therefore, incubate at 22 °C for 3 hours until all enzymes have reached maximum activity, then incubate at 16 °C for 16 to 24 hours to obtain the highest yield. |
|  |  | **5.7)** The competent cells are unhealthy or the procedure for producing competent cells was not performed properly. | **5.7.1)** Utilize a new, healthy, competent cell. |
|  |  | **5.8)** A prolonged incubation at 45⁰C may could result in an increase in the competent cells mortality during transformation in step 28. | **5.8.1)** 60 seconds at 45⁰C or 75 seconds at 42⁰C should be the incubation time. |
| **31** |  | **5.9)** Ampicillin-containing LB broth medium is used in step 29. | **5.9.1)** Use the prewarmed LB Broth medium without ampicillin to give the transformed cells sufficient time to express the ampicillin resistance gene. |
|  |  | **5.10)** LB Agar plates contain a high concentration of the ampicillin. | **5.10.1)** The optimal ampicillin screening concentration should be 100 µg/mL. |
|  | **6)** colonies grow in the negative control. | **6.1)** The digestion process was not performed completely in step 21. | **6.1.1)** Repeat steps 20–21 and load some of this product onto the agarose gel to verify that all vectors have been fully linearized. |
|  | **6)** colonies grow in the negative control. | **6.2)** The ampicillin concentration on LB agar plates is extremely low, allowing non-transformed bacteria to survive or the quality of ampicillin has decreased | **6.2.1)** Use a fresh ampicillin solution and adjust the ampicillin concentration to 100 µg/mL to eradicate non-transformed bacteria. |
| 37&56&73 | **7)** No PCR Product | **7.1)** The annealing temperature is high | **7.1.1)** Use a temperature gradient program, set the temperature 3⁰C to 5⁰C below the lowest T_m_ of the primer pairs, and then select the highest temperature at which the specific product is present. |
|  |  | **7.2)** The concentration of the primer is extremely low | **7.2.1)** Perform PCR reactions according to the master mix instruction. Primers with a final concentration range of 0.2-1 µM are optimal for use. |
|  |  | **7.3)** Use of a DNA template of poor quality. | **7.3.1)** Check the 260/280 ratio of the DNA template with a nanodrop spectrophotometer and analyze the quality of the DNA using gel electrophoresis. If a smear appears, avoid using this template and utilize a new template. |
|  |  | **7.4)** The DNA template may be contaminated with the PCR reaction inhibitor. | **7.4.1)** Ethanol is essential to the DNA extraction protocol's washing phases and acts as a powerful PCR inhibitor. Before eluting the DNA, it is crucial to allow the column's ethanol to evaporate completely. |
|  | **8)** Non-Specific PCR Products | **8.1)** The annealing temperature is low | **8.1.1)** Increase the annealing temperature and try not to decrease the annealing temperature by more than 5°C from the lowest Tm of the primer pairs. |
|  |  | **8.2)** The concentration of the primer is extremely high | **8.2.1)** Reduce the primer concentration and set the final primer concentration to 0.2-1 µM for an optimal PCR reaction. |
|  |  | **8.3)** High concentration of DNA template | **8.3.1)** Use up to 10 ng of plasmid and 1µg of genomic DNA for a 25 µL reaction. |
| **44**  **44** | **9)** A concentration at which the average survival rate becomes zero was not reached  **9)** A concentration at which the average survival rate becomes zero was not reached | **9.1)** The puromycin has expired or was used from an outdated solution. | **9.1.1)** Use a freshly prepared antibiotic solution that has been protected from exposure to light during storage. |
|  |  | **9.2)** The preparation of the culture medium with different concentrations of puromycin in STEP 39 was not done correctly. | **9.2.1)** Repeat the MTT assay using freshly produced culture mediums containing the right amounts of puromycin. |
|  |  | **9.3)** The solutions of puromycin applied directly to the cell-seeded primary culture medium. | **9.3.1)** The direct transfer of puromycin to the wells can lead to errors. It is necessary to discard the cell-seeded culture medium and then add the puromycin-containing medium prepared in step 39. |
|  |  | **9.4)** Cells were seeded in enormous quantities. | **9.4.1)** Seeding a large number of cells causes them to become too dense. This reduces the surface area to volume ratio necessary to absorb an adequate amount of puromycin, which may increase survival. The largest number of cells that can be used in a 96-well plate is 3×10^4^. |
|  | **10)** False negative results after transfection | **10.1)** The treatment time with puromycin had been less than 48 hours. | **10.1.1)** Reducing the treatment time will result in the selection of high puromycin concentrations that may exceed the threshold of transfected cells. Increase the treatment time to 48 hours. |
|  | **11)** False positive results after transfection | **11.1)** The treatment time with puromycin had been more than 72 hours. | **11.1.1)** As the treatment period increases, a concentration of puromycin that can be tolerated by untransfected cells is selected. The treatment period should not exceed 72 hours. |
|  | **12)** Low transfection efficiency or low cell viability after transfection | **12.1)** Too many or too few cells exist at the time of transfection. | **12.1.1)** In both cases, the number of cells can have a negative impact on the final result of transfection. If a medium with 20% FBS is used, the maximum confluence of cells at the time of transfection should not exceed 60%. |
| **51**  **51** |  | **12.2)** The formation of transfection complexes was not performed properly. | **12.2.1)** Dilute lipofectamine and plasmid with Opti-MEM medium. Set the incubation time for each step (46-48) of dilution and preparation of the complex to 30 minutes. |
|  |  | **12.3)** Plasmid concentration is insufficient or plasmids were degraded. | **12.3.1)** Gel electrophoresis must be performed to determine the plasmid's quality. Measure A260/A280 with a nanodrop spectrophotometer; the ratio should be approximately 1.8. |
|  |  | **12.4)** The extracted recombinant plasmids were contaminated with LPS | **12.4.1)** Use an endotoxin removal kit to eliminate LPS contamination, or extract plasmid with a kit that can remove LPS during elution. |
|  |  | **12.5)** Use of penicillin-streptomycin antibiotics during transfection | **12.5.1)** Antibiotics such as penicillin-streptomycin should be avoided during transfection to reduce stress. The increased permeability of cells to antibiotics may result in toxicity. |
|  |  | **12.6)** Transfected cells were treated with puromycin shortly after transfection. | **12.6.1)** After adding the transfection complexes, wait 48 hours then add the antibiotic, which gives the majority of cells time to take up the complex and express the puromycin resistance gene. |
|  |  | **12.7)** Use of a very high concentration of puromycin | **12.7.1)** Extremely high concentrations of puromycin can exceed the tolerance threshold of transfected cells, increasing the mortality of transfected cells. |
|  |  | **12.8)** Prolonging of treatment of puromycin selection beyond 72 hours. | **12.8.1)** Due to the fact that the cells are transiently transfected, and the puromycin resistance gene is temporarily expressed from the plasmid, extending the treatment time can significantly reduce the transfection efficiency. The duration of treatment should not exceed 72 hours. |
|  |  | **12.9)** High-passage number cells were utilized. | **12.9.1)** Increasing the number of cell passages has a negative effect on transfection efficiency. Use cells that have fewer than 10 passages for transfection. |
|  |  | **12.10)** The transfection complexes were added to cells containing serum-free or low serum medium. | **12.10.1)** Transfection efficiency is generally higher in more dynamic cells. Add the transfection complexes to the wells containing a 20% FBS culture medium. |
|  |  | **12.11)** Transfection was done on cells that were not healthy or that were not fully attached to the bottom of the culture plate. | **12.11.1)** The healthy cells seeded in the plate in the appropriate quantity 24 hours before and completely attached to the well should be used for transfection. |
| 56&73 | **13)** Multiple bands in gel electrophoresis result | **13.1)** Long InDels have been occurred. | **13.1.1)** The presence of similar bands in the negative control indicates the presence of contamination. If additional bands were present only in specific cell populations, purify each band separately from the gel and perform Sanger sequencing. |
| 68 | **14)** The isolated single cell cannot grow, or after a few divisions, all the cells die. | **14.1)** Not every cell type will be able to grow under these conditions. Some cell lines do not divide enough in single cell cultures because growth factors secreted by other cells are unavailable. | **14.1.1)** Refer to the critical point in **STEP 66** |
|  |  | **14.2)** The deleted gene may be a crucial gene, such as genes required for embryonic development, whose deletion of them can result in cell death. | **14.2.1)** Perform conditional gene knockout strategy |
