# Supplementary material for: Development of an optimized protocol for generating knockout cancer cell lines using the CRISPR/Cas9 system, with emphasis on transient transfection
Source: PLoS One. 2024 Nov 14;19(11):e0310368. doi: 10.1371/journal.pone.0310368 (PMC11563393; doi:10.1371/journal.pone.0310368)
Supplement: S2 Table — (DOCX) [file pone.0310368.s018.docx]

**S2 Table.** Designed gRNAs using bioinformatics tools for the *EPCAM* gene.

* The gRNA sequence was not found in this tool.
