# Supplementary material for: Development of an optimized protocol for generating knockout cancer cell lines using the CRISPR/Cas9 system, with emphasis on transient transfection
Source: PLoS One. 2024 Nov 14;19(11):e0310368. doi: 10.1371/journal.pone.0310368 (PMC11563393; doi:10.1371/journal.pone.0310368)
Supplement: S5 Table — (DOCX) [file pone.0310368.s021.docx]

**S5 Table.** The distribution pattern of culture medium containing 0.5mg/mL MTT and Puromycin in the 96-well plate
