# Supplementary material for: Development of an optimized protocol for generating knockout cancer cell lines using the CRISPR/Cas9 system, with emphasis on transient transfection
Source: PLoS One. 2024 Nov 14;19(11):e0310368. doi: 10.1371/journal.pone.0310368 (PMC11563393; doi:10.1371/journal.pone.0310368)
Supplement: S9 Table — (DOCX) [file pone.0310368.s025.docx]

**S9 Table.** The average survival rate at different concentrations of puromycin (The selected value is highlighted in grey)
